# West Nile virus infection in the golden hamster (Mesocricetus auratus): a model for West Nile encephalitis.

**DOI:** 10.3201/eid0704.010420

**Published:** 2001

**Authors:** S Y Xiao, H Guzman, H Zhang, A P Travassos da Rosa, R B Tesh

**Affiliations:** Department of Pathology, University of Texas Medical Branch, 301 University Boulevard, Galveston, TX 77555, USA.

## Abstract

This report describes a new hamster model for West Nile (WN) virus encephalitis. Following intraperitoneal inoculation of a New York isolate of WN virus, hamsters had moderate viremia of 5 to 6 days in duration, followed by the development of humoral antibodies. Encephalitic symptoms began 6 days after infection; about half the animals died between the seventh and 14th days. The appearance of viral antigen in the brain and neuronal degeneration also began on the sixth day. WN virus was cultured from the brains of convalescent hamsters up to 53 days after initial infection, suggesting that persistent virus infection occurs. Hamsters offer an inexpensive model for studying the pathogenesis and treatment of WN virus encephalitis.


### West Nile Virus Infection in the Golden Hamster (*Mesocricetus auratus)*: A Model for West Nile Encephalitis

**Shu-Yuan Xiao, Hilda Guzman, Hui Zhang, Amelia P.A. Travassos da Rosa, and Robert B. Tesh**
University of Texas Medical Branch, Galveston, Texas, USA

This report describes a new hamster model for West Nile (WN) virus encephalitis. Following intraperitoneal inoculation of a New York isolate of WN virus, hamsters had moderate viremia of 5 to 6 days in duration, followed by the development of humoral antibodies. Encephalitic symptoms began 6 days after infection; about half the animals died between the seventh and 14th days. The appearance of viral antigen in the brain and neuronal degeneration also began on the sixth day. WN virus was cultured from the brains of convalescent hamsters up to 53 days after initial infection, suggesting that persistent virus infection occurs. Hamsters offer an inexpensive model for studying the pathogenesis and treatment of WN virus encephalitis.

West Nile (WN) virus was first detected in North America in the summer of 1999, during an epidemic and epizootic involving humans, horses, and birds in the New York City metropolitan area (1). The persistence and spread of the virus to several neighboring states during the summer and fall of 2000 suggest that WN virus is now endemic in the United States and that its geographic range probably will continue to expand (2). Although many WN virus infections in humans are asymptomatic or unrecognized, some patients have an acute denguelike illness, and a small percentage have frank meningitis and encephalitis (1-4). The latter complication is most common among the elderly, with recent case-fatality rates ranging from 4% to 11% (3-7).

WN virus is a positive-stranded RNA virus; based on its antigenic and genetic characteristics, it is included in the Japanese encephalitis (JE) serocomplex of the genus *Flavivirus*, family Flaviviridae (8). WN virus was originally isolated from a febrile patient in Uganda in 1937 (9), but it has a worldwide geographic distribution, including most of Africa, southern Europe, central and southern Asia, Oceania, and now North America (3,4,7,10). Despite its wide geographic distribution and frequency, little is known about the pathogenesis of human infection with WN virus, especially encephalitis. One unique pathologic finding in WN virus encephalitis, unlike the encephalitides caused by other closely related flaviviruses, is the targeting of Purkinje cells of the cerebellum (1,7,11-13). We describe preliminary studies of a hamster model for the disease.

### Materials and Methods

Hamsters used in our studies were adult (70 to 100 g) females (*Mesocricetus auratus*) obtained from Harlan Sprague Dawley, Inc. (Indianapolis, IN). They were experimentally infected with a single stock of WN virus strain 385-99. This virus was originally isolated from the liver of a Snowy Owl that died at the Bronx Zoo during the 1999 epizootic in New York City (12); it had been passaged twice in Vero cells.

Virus titrations were done by immunofluorescence (Figure 1) in cultures of C6/36 cells (14) as described (15,16). Blood and 10% brain homogenates were titrated in 24-well tissue culture plates seeded with C6/36 cells. Serial 10-fold dilutions from $10^{-1}$ to $10^{-6}$ were made of each sample in phosphate-buffered saline containing 10% fetal bovine serum (PBS); four wells were inoculated with 0.1 mL of each dilution. The cells were subsequently examined for the presence of WN virus antigen by indirect fluorescent antibody (IFA) test with a WN virus-specific mouse immune ascitic fluid and a commercially prepared (Sigma, St. Louis, MO), fluorescein-conjugated, goat antimouse immunoglobulin (15,16). WN virus titers were calculated as the tissue culture infectious

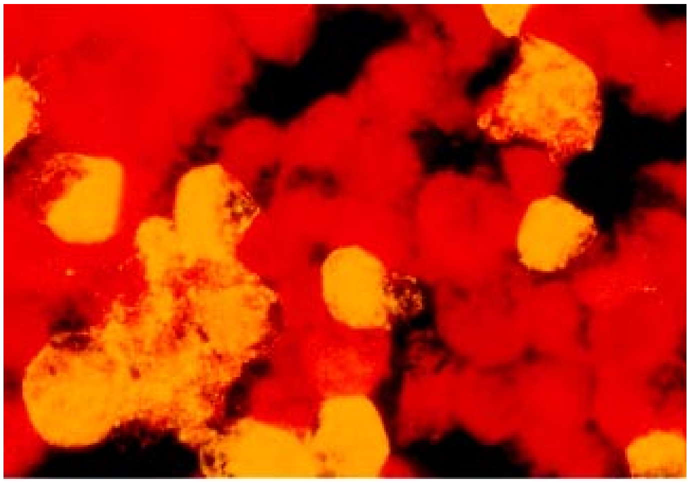

Figure 1. West Nile virus antigen in infected C6/36 cells, as detected by indirect fluorescent antibody testing.

Address for correspondence: Robert B. Tesh, Department of Pathology, University of Texas Medical Branch, 301 University Boulevard, Galveston, Texas 77555-0609 USA; fax: 409-747-2429; e-mail: rtesh@utmb.edu

$dose_{50}$ ($TCID_{50}$) per milliliter of specimen, as described by Reed and Muench (17).

An initial study was done to determine the $ID_{50}$ and the lethal $dose_{50}$ ($LD_{50}$) of WN virus strain 385-99 for adult hamsters. Serial 10-fold dilutions of the virus stock were prepared from $10^{-1}$ to $10^{-6}$ in PBS, and groups of adult hamsters were inoculated intraperitoneally with 0.1 mL of the various dilutions of virus. Hamsters were observed for 28 days, and any deaths were recorded. Brain homogenates from some of the dead animals were inoculated into cultures of Vero or C6/36 cells, which were subsequently examined by IFA to confirm the presence of WN virus. After 4 weeks, serum specimens from surviving hamsters were examined by hemagglutination-inhibition (HI) test for the presence of WN virus antibodies. $LD_{50}$ and $ID_{50}$ values were calculated by the method of Reed and Muench (17). In calculating the $ID_{50}$ of WN virus, both dead and seropositive animals were included. In all subsequent experiments, hamsters were inoculated intraperitoneally with a single virus dose of $10^{4.0}$ $TCID_{50}$.

### Antibody Determinations

Antibodies to WN virus in the infected animals were measured by HI and plaque reduction neutralization tests. Antigens for the HI test were prepared from brains of newborn mice infected with the prototype WN virus strain, B956 (9), by the sucrose-acetone extraction method (18). Hamster sera were tested by HI at serial twofold dilutions from 1:20 to 1:5,120 at pH 6.6, with 4 units of antigen and a 1:200 dilution of goose erythrocytes (18).

The plaque reduction neutralization test was done in microplate cultures of Vero cells as described (19), with a constant virus inoculum (~100 PFU of the Egypt 101 WN virus strain) against varying dilutions of hamster serum. Hamster sera were prepared in twofold dilutions from 1:10 to 1:320 in PBS containing 10% fresh guinea pig serum. The serum-virus mixtures were incubated overnight at 5°C before inoculation. Two microplate wells were inoculated with each serum dilution. Plaques were read on the sixth day; samples producing $\geq$90% plaque reduction were considered positive.

### Histologic Examination of Tissues

Under Halothane (Halocarbon Laboratories, River Edge, NJ) anesthesia, hamsters were exanguinated by cardiac puncture. The chest cavity was opened quickly, and 20 to 30 mL of 10% buffered formalin was injected directly into the left ventricle to perfuse the body. After refrigeration overnight at 5°C, the body was dissected, and samples of lung, liver, spleen, pancreas, kidney, and spinal cord, as well as the entire brain, were removed and placed in 10% buffered formalin solution for another 24 hours to allow proper fixation. The following day tissue samples were transferred to 70% ethanol for storage. These specimens were subsequently processed, and histologic slides were prepared as described (20). Special stains (Luxol Fast Blue and Nissl's) were also performed on some brain and spinal cord sections. Tissues from six uninfected hamsters were fixed and processed by the same techniques; these tissues were included as controls in all histologic and immunohistochemical examinations.

### Immunohistochemical Detection of WN Viral Antigen

After deparaffinization, the formalin-fixed, paraffin-embedded tissue sections (3- to 4-µm thick) were immersed in 3% $H_2O_2$ for 10 minutes to block endogenous peroxidase activity. This was followed by an antigen retrieval heating step, with a citrate buffer (10% target retrieval solution, DACO, Carpinteria, CA), at 90°C for 30 minutes. A WN virus immune ascitic fluid was used as the primary antibody at a dilution of 1:100. A commercially available mouse-on-mouse immunostain kit (InnoGenex, San Ramon, CA) was used to detect specifically bound primary antibodies and prevent nonspecific binding between species (20).

### In Situ TUNEL Assay

The terminal deoxynucleotidyl transferase-mediated dUTP nick-end labeling (TUNEL) technique was used to assay for apoptosis in stained sections of hamster brain and spinal cord, with the ApopTag peroxidase kit (Intergen Company, Purchase, NY) and 3,3'-diaminobenzidine as the chromogen (20). Slides were counterstained with hematoxylin and mounted with a cover slip for microscopic examination. Different regions of the section were evaluated individually. For semiquantitative assessment of the apoptotic activity, a single 20x-objective microscopic field was selected that contained the highest number of positively stained cells. The activity was scored as follows: 0 = no positive cells; 1 =  5 positive cells; 2 = 6 to 10 positive cells; 3 = 11 to 20 positive cells; and 4 = >20 positive cells.

### Results

An initial experiment was carried out to determine the $ID_{50}$ and $LD_{50}$ of the 385-99 WN virus stock in adult female hamsters following intraperitoneal inoculation (Table 1). The hamster $ID_{50}$ of the virus stock was estimated to be $10^{-6.3}$/mL (17), but the $LD_{50}$ was difficult to calculate because the percentage of deaths at various dilutions did not give a clear endpoint. A similar irregular pattern of death or encephalitis, compared with the $ID_{50}$, was also reported in hamsters experimentally infected with tick-borne encephalitis (TBE) virus (21). Based on these results, $10^{4.0}$ $TCID_{50}$ was selected as the infecting dose of WN virus to be used in subsequent hamster experiments.

The pattern of illness in the hamsters in all experiments was similar. During the first 5 days after infection, the hamsters appeared normal. At day 6 or 7, the animals became lethargic and remained huddled together in the corners of their cages. Food and water consumption by the animals decreased, as did grooming activity. At days 7 to 10, many of the animals had neurologic symptoms, including hind limb paralysis, tremors, difficulty in walking, circling, and loss of

Table 1. Mortality and infection rates among adult hamsters following intraperitoneal inoculation of serial 10-fold dilutions of West Nile virus strain 385-99

| Virus titer of inoculum[a] | No inoculated | No. infected (%) | No. dead (%) |
|---|---|---|---|
| $10^{6.0}$ | 10 | 10 (100) | 5 (50) |
| $10^{5.0}$ | 10 | 10 (100) | 7 (70) |
| $10^{4.0}$ | 10 | 10 (100) | 6 (60) |
| $10^{3.0}$ | 10 | 9 (90) | 6 (60) |
| $10^{2.0}$ | 10 | 8 (80) | 2 (20) |
| $10^{1.0}$ | 10 | 8 (80) | 2 (20) |
| $10^{0.0}$ | 7 | 1 (14) | 0 (0) |

[a]Tissue culture infectious $dose_{50}$ ($TCID_{50}$) as determined by titration in mosquito cell (C6/36) cultures.

balance. Many of the severely affected animals died 7 to 14 days after infection. Animals still alive at 14 days usually survived, although some had residual neurologic signs (tremors, muscle weakness, and difficulty in walking).

### Viremia and Antibody Response Following WN Virus Infection of Hamsters

Figure 2 shows the pattern of viremia and immune response in 10 adult hamsters following intraperitoneal inoculation of $10^4$ $TCID_{50}$ of WN virus strain 385-99. These animals were bled daily for 7 days. Moderate levels of viremia were detected in the hamsters within 24 hours after infection; viremia persisted for 5 or 6 days. HI antibodies were detected in all the animals by day 5, and the titers continued to increase through day 7 (Table 2). No infectious virus was detected in the blood after day 6. The same pattern was observed regardless of outcome (i.e., fatal encephalitis or recovery). Titration of 10% brain suspensions of hamsters that developed clinical encephalitis during the second week of infection yielded virus titers ranging from $10^3$ to $10^{6.5}$ $TCID_{50}$, although no infectious virus was detected in peripheral blood at this time and high titers of HI and neutralizing antibodies were present in sera.

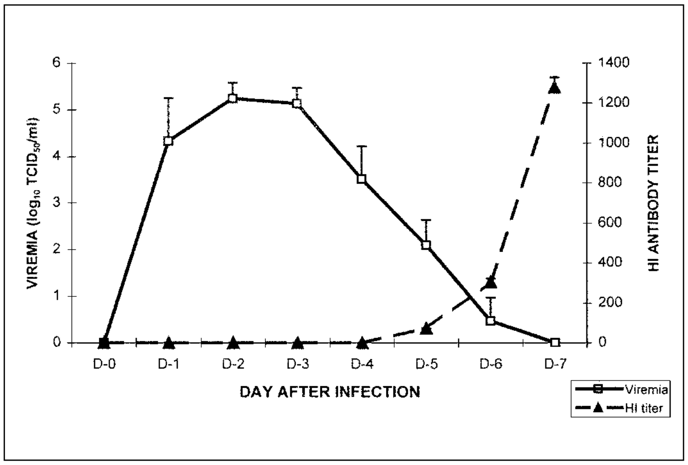

Figure 2. Daily mean (+ standard deviation) virus titers and hemagglutination inhibition antibody levels in 10 hamsters following intraperitoneal inoculation of $10^4$ $TCID_{50}$ of West Nile virus strain 385-99.

Table 2. Viremia and hemagglutination inhibition (HI) antibody response in 10 adult hamsters following intraperitoneal inoculation of $10^4$ $TCID_{50}$[a] of West Nile virus strain 385-99

| Animal no. | Day 1 | Day 2 | Day 3 | Day 4 | Day 5 | Day 6 | Day 7 |
|---|---|---|---|---|---|---|---|
| 8001 | 4.3(N)[b] | 5.0(N) | 5.0(N) | 3.3(N) | 1.0(80) | 1.0(320) | <0.7(640) |
| 8002 | 4.7(N) | 5.5(N) | 5.2(N) | 3.5(N) | 2.0(80) | <0.7(320) | <0.7(640) |
| 8003 | 5.3(N) | 5.3(N) | 5.0(N) | 3.5(N) | 2.5 (40) | <0.7(320) | <0.7(NT) |
| 8004 | 2.0(N) | 5.0(N) | 5.0(N) | 4.3(N) | 2.5(40) | 1.0(160) | <0.7(640) |
| 8005 | 4.0(N) | 5.0(N) | 5.5(N) | 3.7(N) | 1.7(80) | 1.0(320) | <0.7(1280) |
| 8006 | 4.6(N) | 5.2(N) | 5.7(N) | 4.3(N) | 2.7(80) | <0.7(320) | <0.7(2560) |
| 8007 | 4.3(N) | 5.7(N) | 4.6(N) | 4.0(N) | 2.0(80) | 1.0(320) | <0.7(2560) |
| 8008 | 4.2(N) | 5.8(N) | 4.8(N) | 1.8(N) | 2.0(80) | <0.7(320) | <0.7(640) |
| 8009 | 5.2(N) | 5.2(N) | 5.0(N) | 3.2(N) | 2.8(80) | <0.7(320) | <0.7(1280) |
| 8010 | 4.7(N) | 4.7(N) | 5.5(N) | 3.5(N) | 1.8(80) | 0.7(320) | <0.7(1280) |

[a]$TCID_{50}$ = tissue culture infectious $dose_{50}$
[b]Level of viremia expressed as $log_{10}$ $TCID_{50}$ of virus/mL of blood. (reciprocal of HI antibody titer) N = < 1:20.

### Pathologic Findings in Hamsters with WN Virus Encephalitis

In a third experiment, 60 adult hamsters were inoculated intraperitoneally with $10^4$ $TCID_{50}$ of WN virus strain 385-99. Beginning on day 1 postinfection, two hamsters were killed daily for 10 consecutive days; these animals were perfused with 10% formalin to fix tissues for histologic study. Some surviving animals from this experiment were subsequently examined for persistent WN virus infection, as described later.

Histopathologic examination of liver, kidney, lung, myocardium, pancreas, and spleen of the infected hamsters during this initial 10-day period showed no substantial pathologic changes, except for spotty splenic necrosis in a few animals. In contrast, substantial, progressive pathologic changes were observed in the brain and spinal cord.

During the first 4 days, no discernible pathologic changes were observed in the brain or spinal cord. Beginning on day 5, however, neuronal degeneration was seen in many areas, manifested by shrinkage of the perikaryon with intense eosinophilia of the cytoplasm, central chromatolysis, and condensation of the nucleus. Small clusters of large neurons undergoing these changes were located in the cerebral cortex, cerebellar cortex (Purkinje cells), and subcortical gray matter; the hippocampus and basal ganglia were also affected, but less severely. No abnormalities were seen in the spinal cord at this stage. These changes became more extensive on day 6, with involvement of both the superficial and deeper layers of the cerebral cortex, as well as the hippocampus (Figure 3). Large neurons adjacent to the olfactory bulb also showed similar degeneration. Likewise, Purkinje cell degeneration in the cerebellum became more severe and extensive, and scattered degenerating neurons began to appear in the brain stem. At this stage, no inflammatory cell infiltration or perivascular inflammation was seen except for perivascular edema. However, mild perivascular inflammation was observed in the spinal cord. By days 7 and 8 postinfection, neuronal degeneration became more localized, mainly involving the deeper layers of the cerebral cortex, occasional large neurons in the olfactory nucleus, scattered Purkinje cells in the cerebellum (Figure 3), and the brain stem. At this stage, ill-formed microglial nodules and minimal perivascular inflammation began to appear. The microglial nodules consisted of small microglial cells surrounding degenerative neurons (Figure 3). Mild perivascular inflammation with focal neuronal degeneration was observed in the spinal cord, mainly involving the anterior horn. On day 9, more neuronal degeneration, along with psammoma bodies, was seen in the olfactory nucleus; changes in spinal cord were similar to those on day 8. On day 10, most of the abnormalities were localized in the brain stem, which exhibited focal neuronal degeneration surrounded by microglial cell infiltration and "spongiform" neuropil (Figure 3F). Inflammation in the spinal cord was diffuse.

Brains from some surviving hamsters were also examined pathologically on days 12, 14, 19, 28, 35, and 48 after infection. Microscopic changes observed included focal loss of Purkinje cells, occasional microglial clustering, and psammoma bodies.

### Immunohistochemical Detection of WN Virus Antigen

WN virus antigen was not detected in the brain during the first 5 days after inoculation. On day 6, clusters or

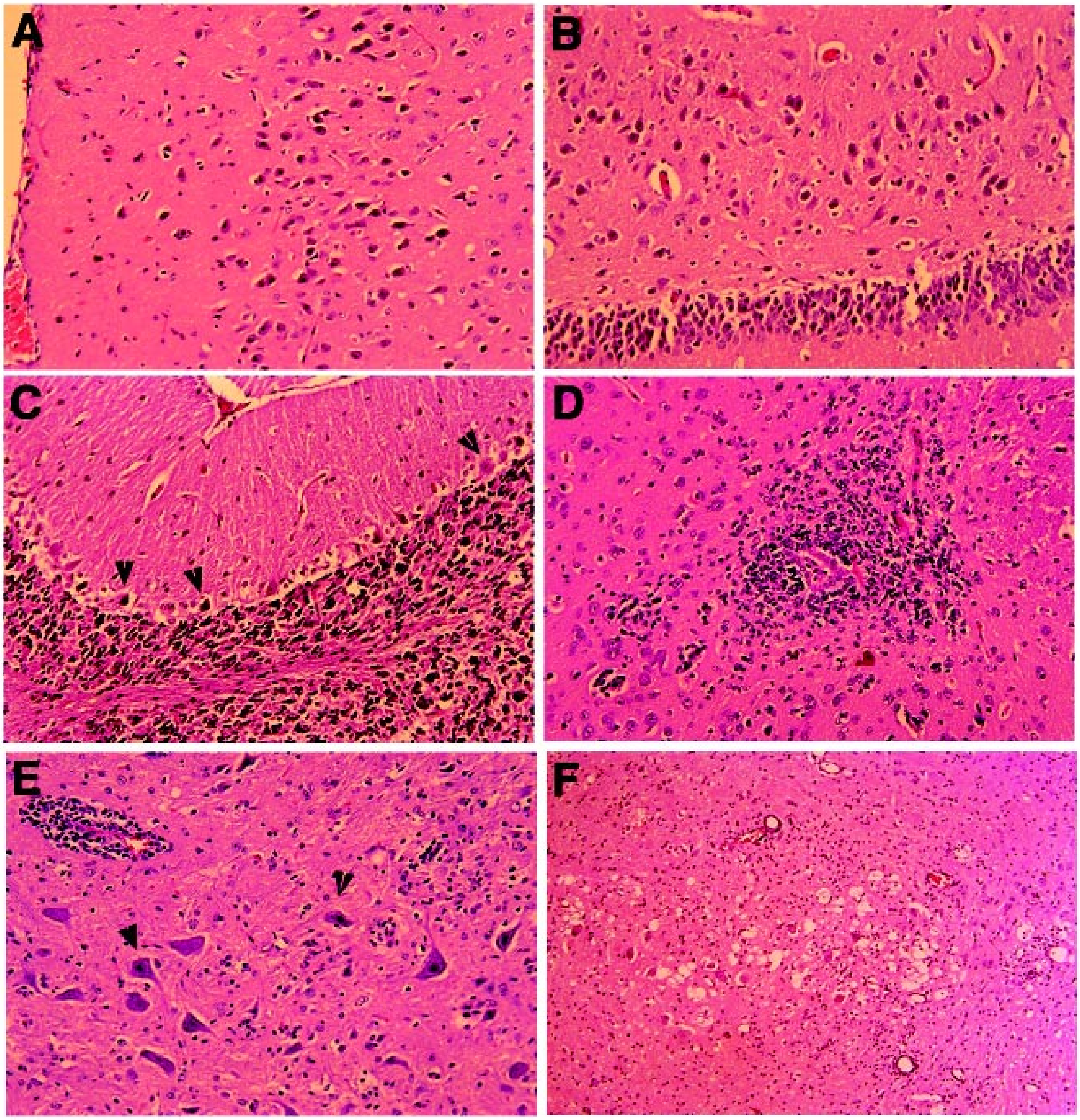

Figure 3. Histologic changes in brains of West Nile virus-infected hamsters. a. Cerebral cortex, with many degenerating neurons, day 6 postinfection. b. Hippocampus, showing large neurons undergoing degeneration, day 6. c. Cerebellar cortex, with frequent Purkinje call degeneration (shrunken cells, arrowheads) and loss, day 8. d. A microglial nodule near blood vessel in basal ganglia, day 9. e. Mild perivascular inflammation (upper left field), neurons with nuclear condensation (arrowhead) and cytoplasmic eosinophilia, in brain stem, day 9. f. Spongiform change in the brain stem, day 10. Magnification 100x.

individual neurons with antigen-positive cytoplasmic staining were observed in the basal ganglia and the brain stem (Figure 4). Other regions of the brain were negative. By day 7, the amount of antigen had increased, and antigen appeared in neurons of the cerebellar cortex, subcortical gray matter, brain stem, basal ganglia, and, to a lesser degree, in the frontal and parietal cortices and the hippocampus. In the cerebrum, foci of positive cells were more prominent immediately adjacent to the ventricles. The amount of viral antigen detected on days 8 and 9 decreased. By day 10 of infection, antigen appeared focally (but strongly) only in the brain stem (Figure 4). None of the hamsters had WN virus antigen in the olfactory bulb by immunohistochemical staining.

Spinal cord sections from two hamsters were stained immunohistochemically on day 9 postinfection. One or two large neurons from each side of the anterior horn were positive for WN virus antigen. The positive neurons were limited in number but were present at most of the spinal levels examined, particularly in the thoracic and lumbar regions.

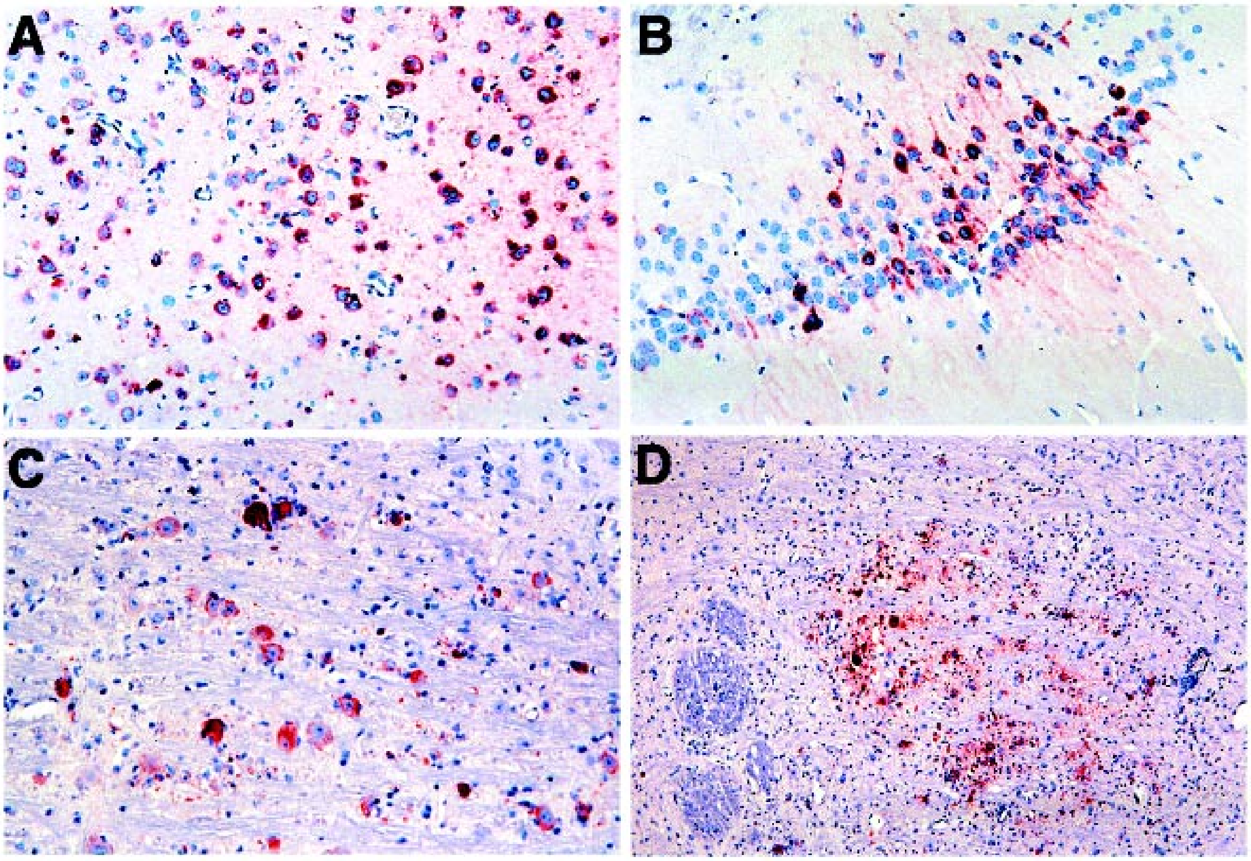

Figure 4. Immunohistochemical detection of West Nile virus antigen in brains of inoculated hamsters. The photomicrographs demonstrate strong cytoplasmic staining (red color) of large and small neurons in different regions. a. Cerebral cortex, day 8 postinfection. b. Hippocampus, day 7. c. Basal ganglia, day 7. d. Brain stem, day 10. Magnification: a-c 100x; d 50x.

### In Situ TUNEL Assay

The TUNEL assay, which selectively stains apoptotic cells, was performed on both brain and spinal cord (22). Apoptotic cells were observed in all the areas where neuronal degeneration was seen histologically, but positive-staining cells were most prominent in the hippocampus and basal ganglia (Figure 5).

Brain sections from two or three hamsters from each day postinfection were studied by this method (Figure 6). Rare apoptotic cells began to appear on day 6, and activity gradually increased and peaked on day 9. The positive cells were not limited to neurons but included some endothelial cells of blood vessels in the same microscopic fields. In general, the concentration of apoptotic cells appeared to be most intense in the basal ganglia and brain stem. High activity also appeared transiently in the cerebellar cortex, including both Purkinje cells and scattered medium-sized neurons in the deeper levels (Figure 6).

### Persistent WN Virus Infection in Hamsters

Because of an earlier report (23) that WN virus persisted for up to 5½ months in the brains of experimentally infected monkeys, we investigated this possibility in hamsters. Eleven animals that survived intraperitoneal inoculation of WN virus in the third experiment were killed at intervals of 19, 27, 35, 42, and 52 days after infection. A blood sample was taken before death for culture and antibody determinations, and a portion of the cerebellum was also removed at necropsy for culture. WN virus was recovered in Vero cell cultures inoculated with brain homogenates from 5 of 11 convalescent hamsters sampled. WN virus was recovered from one of two hamsters killed on day 19, one of two on day 27, one of two on day 35, one of two on day 42, and one of three on day 52. The positive cultures showed typical WN virus cytopathic effect (CPE) and were confirmed by IFA with a WN virus immune ascitic fluid. IFA of Vero cells from brain cultures without CPE gave negative results. An attempt was made to titrate some of the persistently infected brain samples in C6/36 cells, but the titers were very low ($<10^{0.7}$ to $10^{1.0}$ $TCID_{50}$/mL of 10% brain suspension). Blood cultures of the same hamsters were negative, and the sera had high titers of HI and neutralizing antibodies.

### Conclusion

The sequence of events following intraperitoneal inoculation of WN virus into adult hamsters was similar to that described in experimental studies of other flavivirus encephalitides (11,23-27). After a brief viremia of 5 to 6 days' duration, humoral antibodies developed (Figure 2). During this period, the hamsters were asymptomatic. Beginning

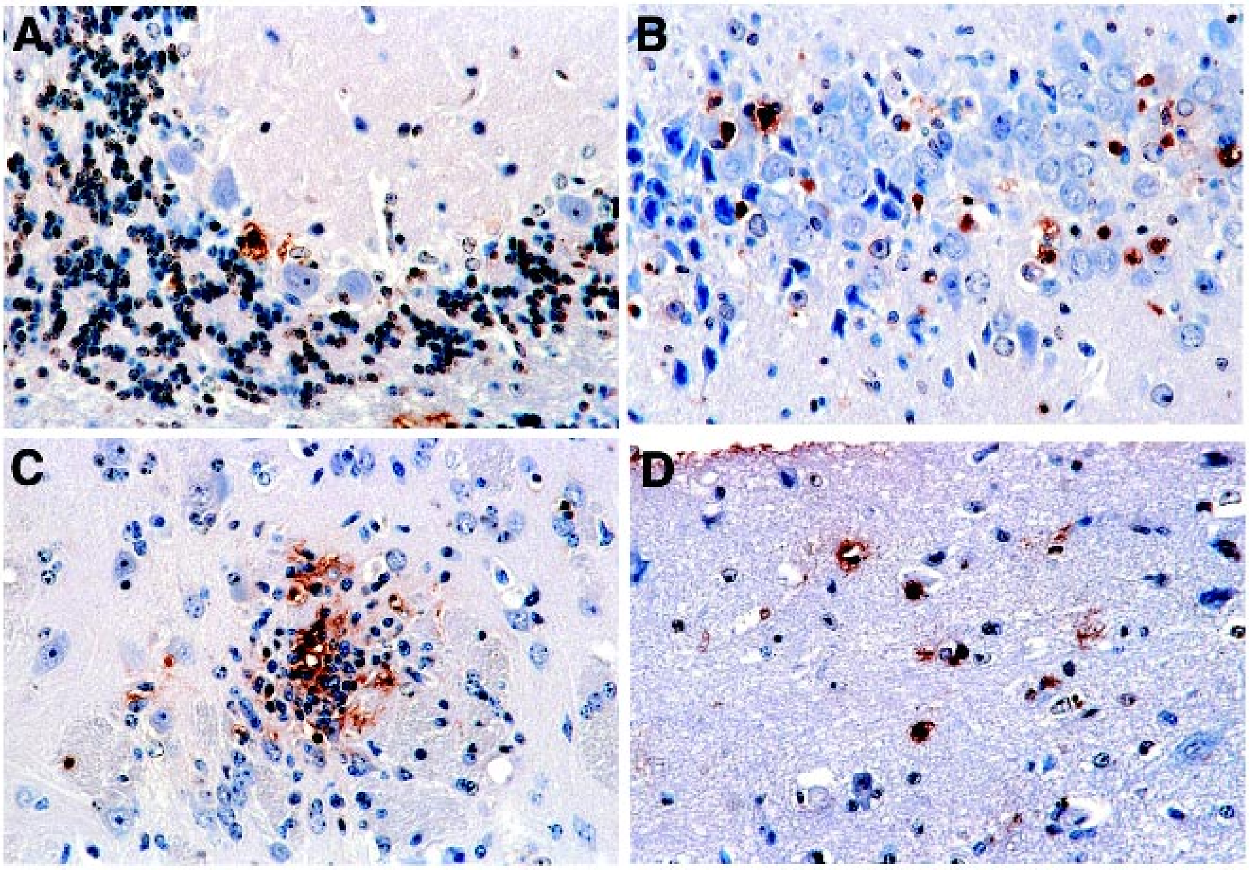

Figure 5. In situ terminal deoxynucleotidyl transferase-mediated dUTP nick-end labeling (TUNEL) staining (ApopTaq peroxidase kit, Intergen Company, Purchase, NY) of neurons undergoing apoptotic cell death (brown-colored nuclear staining). a. Cerebellar cortex, showing occasional positively stained Purkinje cells. b. Hippocampus. c. Positively stained neurons within a microglial nodule. d. Positively stained neurons in cerebral cortex. Magnification 200x.

day 6 postinfection, many of the animals had clinical signs of acute central nervous system (CNS) injury (somnolence, muscle weakness, paralysis, tremors, and loss of balance) with a substantial number of deaths occurring on days 7 to 14. Histologically, neuronal degeneration in the brain also was not seen until day 6 after infection. The histopathologic changes began in the cerebral cortex, involving all layers, but gradually only the deeper layers were involved. The observed histopathologic changes eventually spread to the basal ganglia, hippocampus, cerebellar cortex (as Purkinje cell degeneration and loss), and brain stem. At first, neuronal degeneration was not accompanied by microglial cell infiltration or perivascular inflammation. These processes appeared later, with well-formed microglial nodules, sometimes containing a degenerating neuron at the center. The in situ TUNEL analysis confirmed that many of the degenerating neurons underwent apoptosis, leading to the loss of these neurons. This observed sequence suggests that WN virus entered the brain and infected neurons first and that the inflammatory infiltration (perivascular inflammation and microgliosis) was a secondary response to neuronal damage caused by the virus. Supporting this observation is the fact that both histologic abnormalities and appearance of viral antigen (see below) were observed in the brain first and spinal cord second.

Using immunohistochemical staining, WN virus antigen was first detected in the brain on day 6 after infection. This timing correlated well with the onset of encephalitic

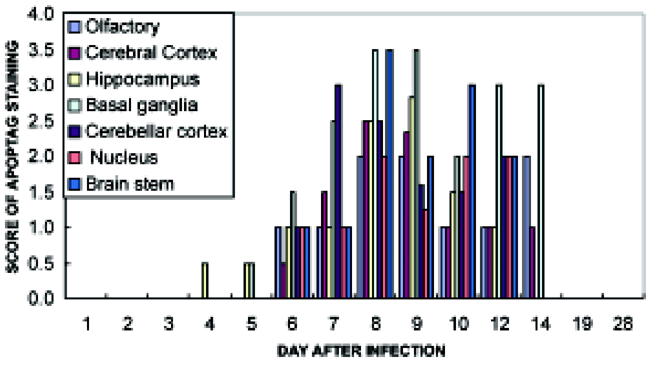

Figure 6. Semiquantitative analysis of ApopTaq-labeled cells in different areas of the brain. Each area was scored individually on a scale of 0 to 4 for positive staining intensity (see Materials and Methods).

symptoms and the observed histopathology in the infected hamsters. Viral antigen was detected in all the areas that showed histologic lesions on routine hematoxylin and eosin (H&E) staining, including cerebral cortex, deep cerebral nuclei, hippocampus, basal ganglia, cerebellar cortex, and the brain stem. Antigen positivity persisted longer in the brain stem. The distribution of the WN virus antigen-positive cells in the brain was focal; these cells usually formed discrete clusters rather than a diffuse pattern. This focal distribution may be caused by regional differences in blood-brain barrier integrity or differential sensitivity of neurons. By day 10, viral antigen was no longer detectable except focally in the brain stem; it completely disappeared afterwards. No viral antigen was detected in the olfactory nucleus in any of the animals examined.

Despite frequent foci of WN virus antigen positivity and neuronal degeneration, inflammatory cell infiltration (i.e., microglial nodules) was not prominent in the hamsters. As shown by the in situ TUNEL analysis, many of the cells undergoing apoptosis were not associated with inflammatory cell attack. This observation suggests that cell death, caused directly by WN virus infection, is the main mechanism of neuronal damage. The exact mechanism by which WN virus initiates the cell death pathway is not clear and will be the subject of future studies. However, experimental studies with Sindbis virus (genus *Alphavirus,* family Togaviridae) (28) and neurovirulent dengue viruses (genus *Flavivirus*) (29) indicate that these mosquito-borne viruses also cause encephalitis by inducing neuronal apoptosis.

Many earlier experimental studies of WN encephalitis were done in monkeys or mice (11,23,24,30-34). Monkeys are no longer a viable option for most investigators because of their cost and the regulatory issues involved in their use. The histopathologic changes reported in the brain and spinal cord of parenterally infected adult mice (24,31,32) are similar to those observed in the WN virus-infected hamsters. However, in preliminary studies with outbred adult Institute for Cancer Research mice, we observed that the New York strain was highly lethal by the intraperitoneal route, but that the viremia following infection was minimal. Other investigators have reported similar results with WN virus strains of Middle Eastern or African origin (24,30,31,33). For this reason, we decided to use hamsters as our animal model, since infection in this rodent species seemed more similar to infection in humans and horses (3,7).

Pogodina et al. (23,34) reported that both WN and tick-borne encephalitis (TBE) viruses induce persistent infection in the CNS of experimentally infected rhesus monkeys, regardless of the route of inoculation or the symptoms (overt or asymptomatic) of the acute infection. These investigators showed that WN virus could be detected for up to 5½ months in the CNS of monkeys after initial infection and that TBE virus could be detected for up to 783 days after infection by cocultivation of trypsinized brain cells on a monolayer of indicator cells (23,34-36). Furthermore, the viruses recovered from the persistently infected monkey brains differed in their phenotypic characteristics (37). The aforementioned monkey experiments were done more than 20 years ago, and the phenotypically altered viruses were not characterized genetically. However, our recovery of WN virus from the brains of persistently infected hamsters supports this earlier Russian work.

The studies of WN virus persistence in the brains of experimentally infected hamsters were not carried beyond 52 days, so the duration and eventual outcome of chronic CNS infection in the animals are unknown. WN virus was recovered from the brains of 5 of 11 convalescent hamsters; but in retrospect, the method of virus assay used (direct culture of a crude brain homogenate) was probably not optimal. Most of the surviving hamsters had WN virus-neutralizing antibody titers $\geq$1:320 when tested 1-2 months after infection. Since a homogenate of brain tissue inevitably contains traces of blood that are present in small vessels, antibodies in the blood may reduce the sensitivity of this culture method (38). Consequently, the cocultivation technique (36) of Pogodina et al. (35) or reverse-transcription polymerase chain reaction would seem preferable, since these assay methods reduce the inhibiting effect of antibodies.

Evidence of persistent infection and chronic progressive neurologic disease following flavivirus encephalitis has been described, especially with Japanese encephalitis and members of the TBE complex (25,39-44). The mechanism and sequelae of persistent CNS infection by flaviviruses are poorly understood but may be of considerable public health importance in the light of the frequency of human infection with some of these agents. Our preliminary results suggest that WN virus infection in hamsters may be a useful experimental model for persistent flavivirus CNS infection. The hamster also provides a reliable and inexpensive animal model for study of the pathogenesis and treatment of WN virus encephalitis.

### Acknowledgments

We thank Tracy McNamara for providing the 385-99 virus strain and Dora Salinas for help in preparing the manuscript.

This work was supported by National Institutes of Health grants AI-10984 and AI-39800.

Dr. Xiao is Associate Professor in the Department of Pathology at the University of Texas Medical Branch, where he is also a member of the Center for Tropical Diseases.

### References

1. Centers for Disease Control and Prevention. Update: West Nile-like viral encephalitis—New York, 1999. MMWR Morb Mortal Wkly Rep 1999;48:890-2.
2. Centers for Disease Control and Prevention. Update: West Nile virus activity—eastern United States, 2000. MMWR Morb Mortal Wkly Rep 2000;49:1044-7.
3. Hayes CG. West Nile fever. In: Monath TP, editor. The arboviruses: epidemiology and ecology. Vol 5. Boca Raton (FL): CRC Press; 1989. p. 60-82.
4. Hubalek Z, Halouzka J. West Nile fever—a reemerging mosquito-borne viral disease in Europe. Emerg Infect Dis 1999;5:643-50.
5. Asnis DS, Conetta R, Teixeira AA, Waldman G, Sampson BA. The West Nile virus outbreak of 1999 in New York: the Flushing Hospital experience. Clin Infect Dis 2000;30:413-8.
6. Tsai TF, Popovici F, Cernescu C, Campbell GL, Nedelcu NI. West Nile encephalitis epidemic in southeastern Romania. Lancet 1998;352:767-71.
7. Komar N. West Nile encephalitis. Rev Sci Tech 2000;19:166-76.
8. Heinz FX, Collett MS, Purcell RH, Gould EA, Howard CR, Houghton M, et al. Family Flaviviridae. In: van Regenmortel MHV, Fauquet CM, Bishop DHL, Carstens EB, Estes MK, Lemon SM, et al., editors. Virus taxonomy: classification and nomenclature of viruses. San Diego: Academic Press; 2000. p. 859-78.

9. Smithburn KC, Hughes T, Burke A, Paul J. A neurotropic virus isolated from the blood of a native of Uganda. Am J Trop Med Hyg 1940;20:471-8.
10. Taylor RM, Work TH, Hurlbut HS, Rizk F. A study of the ecology of West Nile virus in Egypt. Am J Trop Med Hyg 1956;5:579-620.
11. Manulidis EE. Neuropathology of experimental West Nile virus infection in monkeys. J Neuropath Exp Neurol 1956;15:448-60.
12. Steele KE, Linn MJ, Schoepp RJ, Komar N, Geisbert TW, Manduca RM, et al. Pathology of fatal West Nile virus infections in native and exotic birds during the 1999 outbreak in New York City, New York. Vet Pathol 2000;37:208-24.
13. Sampson BA, Ambrosi C, Charlot A, Reiber K, Veress JF, Armbrustmacher V. The pathology of human West Nile virus infection. Hum Pathol 2000;31:527-31.
14. Igarashi A. Isolation of a Singh's *Aedes albopictus* cell clone sensitive to dengue and chikungunya viruses. J Gen Virol 1978;40:531-44.
15. Tesh RB. A method for the isolation of dengue viruses, using mosquito cell cultures. Am J Trop Med Hyg 1979;28:1053-9.
16. Tesh RB, Guzman H, Travassos da Rosa APA, Vasconcelos PFC, Dias LB, Bunnell JE, et al. Experimental yellow fever virus infection in the golden hamster (*Mesocricetus auratus*). 1. Virologic, biochemical and immunologic studies. J Infect Dis 2001;183:1431-36.
17. Reed LJ, Muench H. A simple method of estimating fifty percent endpoints. Am J Hyg 1938;27:493-7.
18. Beaty BJ, Calisher CH, Shope RE. Arboviruses. In: Schmidt NJ, Emmons RW, editors. Diagnostic procedures for viral, rickettsial and chlamydial infections, 6th ed. Washington: American Public Health Association; 1989. p. 797-856.
19. Tesh RB, Peters CJ, Meegan JM. Studies on the antigenic relationship among phleboviruses. Am J Trop Med Hyg 1982;31:149-55.
20. Xiao S-Y, Zhang H, Guzman H, Tesh RB. Experimental yellow fever virus infection in the golden hamster (*Mesocricetus auratus*). 2. Pathology. J Infect Dis 2001;183:1437-44.
21. Slonim D, Zavadova H, Simon J. Pathogenicity of tick-borne encephalitis virus. 4. Relation between infective and pathogenic activity for golden hamsters. Acta Virol 1966;10:336-42.
22. Lemasters JJ. Mechanisms of hepatic toxicity. V. Necrapoptosis and mitochondrial permeability transition: shared pathways to necrosis and apoptosis. Am J Physiol 1999;276:G1-G6.
23. Pogodina VV, Frolova MP, Malenko GV, Fokina GI, Koreshkova GV, Kiseleva LL, et al. Study on West Nile virus persistence in monkeys. Arch Virol 1983;75:71-86.
24. Nathanson N. Pathogenesis. In: Monath TP, editor. St. Louis encephalitis. Washington: American Public Health Association; 1980. p. 201-36.
25. Monath TP, Heinz FX. Flaviviruses. In: Fields BN, Knipe DM, Howley PM, editors. Fields virology. 3rd ed. Vol 1. Philadelphia: Lippincott-Raven; 1996. p. 961-1034.
26. Monath TP, Cropp CB, Harrison AK. Mode of entry of a neurotropic arbovirus into the central nervous system. Reinvestigation of an old controversy. Lab Invest 1983;48:399-410.
27. McMinn PC, Dalgarno L, Weir R. A comparison of the spread of Murray Valley encephalitis viruses of high or low neuroinvasiveness in the tissues of Swiss mice after peripheral inoculation. Virology 1996;220:414-23.
28. Lewis J, Wesselingh SL, Griffin DE, Hardwick JM. Alphavirus-induced apoptosis in mouse brains correlates with neurovirulence. J Virol 1996;70:1828-35.
29. Despres P, Frenkiel M-P, Ceccaldi P-E, Duarte dos Santos C, Deubel V. Apoptosis in the mouse central nervous system in response to infection with mouse-neurovirulent dengue viruses. J Virol 1998;72:823-9.
30. Eldadah AH, Nathanson N, Sarsitis R. Pathogenesis of West Nile virus encephalitis in mice and rats. I. Influence of age and species on mortality and infection. Am J Epidemiol 1967;86:765-75.
31. Weiner LP, Cole GA, Nathanson N. Experimental encephalitis following peripheral inoculation of West Nile virus in mice of different ages. J Hyg 1970;68:435-46.
32. Eldadah AH, Nathanson N. Pathogenesis of West Nile encephalitis in mice and rats. II. Virus multiplication, evolution of immunofluorescence, and development of histological lesions in the brain. Am J Epidemiol 1967;86:776-90.
33. Lustig S, Olshevsky U, Ben-Nathan D, Lachmi B-E, Malkinson M, Kobiler D, et al. A live attenuated West Nile virus strain as a potential veterinary vaccine. Viral Immunol 2000;13:401-10.
34. Pogodina VV, Frolova MP, Malenko GV, Fokina GI, Levina LS, Mamonenko LL, et al. Persistence of tick-borne encephalitis virus in monkeys. I. Features of experimental infections. Acta Virol 1981;25:337-43.
35. Pogodina VV, Malenko GV, Fokina GI, Levina LS, Koreshkova GV, Rzhakhova OE, et al. Persistence of tick-borne encephalitis virus. II. Effectiveness of methods used for virus detection. Acta Virol 1981;25:344-51.
36. Shope TC, Klein-Robbenhaar J, Miller G. Fatal encephalitis due to *Herpesvirus hominis*: use of intact brain cells for isolation of virus. J Infect Dis 1972;125:542-4.
37. Pogodina VV, Levina LS, Fokina GI, Koreshkova GV, Malenko GV, Bochkova NG, et al. Persistence of tick-borne encephalitis virus in monkeys. 3. Phenotypes of the persisting virus. Acta Virol 1981;25:352-60.
38. Rajcani J, F. Ciampor SA, Likikova H, Rosenbergova M. Activation of latent *Herpesvirus hominis* in explants of rabbit trigeminal ganglia: the influence of immune serum. Arch Virol 1977;53:55-69.
39. Levy JP, Silvestre D, LeClerc JC, Boiron M. Chronic disease and virus persistence in mice inoculated with Kysanur Forest disease virus. Virology 1966;29:679-83.
40. Zlotnik I, Carter GB, Grant DP. The persistence of louping ill virus in immunosuppressed guinea pigs. Br Exp Pathol 1971;52:395-407.
41. Gresikova M, Calisher CH. Tick-borne encephalitis. In: Monath TP, editor. The arboviruses: epidemiology and ecology. Vol 4. Boca Raton (FL): CRC Press; 1989. p. 177-202.
42. Gaidamovich S Ya. Tick-borne *Flavivirus* infection. In: Porterfield JS, editor. Exotic viral infections. London: Chapman and Hall; 1995. p. 203-21.
43. Ilienko VI, Komandenko NI, Platonov VG, Prozorova IN, Panov AG. Pathogenetic study on chronic forms of tick-borne encephalitis. Acta Virol 1974;18:341-6.
44. Zlotnik I, Grant DP, Carter GB. Experimental infection of monkeys with viruses of the tick-borne encephalitis complex: degenerative cerebellar lesions following inapparent forms of the disease or recovery from clinical encephalitis. Br J Exp Pathol 1976;57:200-10.
